# Nioboaeschynite-(Ce), Ce(NbTi)O_6_

**DOI:** 10.1107/S1600536812031765

**Published:** 2012-07-18

**Authors:** Shaunna M. Morrison, Robert T. Downs, Kenneth J. Domanik, Hexiong Yang, Donald Doell

**Affiliations:** a Department of Geosciences, University of Arizona, 1040 E. 4th Street, Tucson, Arizona 85721-0077, USA; b Lunar and Planetary Laboratory, University of Arizona, 1629 E. University Boulevard, Tucson, AZ 85721-0092, USA; c 122 Dublin Street, Peterborough, Ontario, K9H 3A9, Canada

## Abstract

Nioboaeschynite-(Ce), ideally Ce(NbTi)O_6_ [cerium(III) niobium(V) titanium(IV) hexa­oxide; refined formula of the natural sample is Ca_0.25_Ce_0.79_(Nb_1.14_Ti_0.86_)O_6_], belongs to the aeschynite mineral group which is characterized by the general formula *AB*_2_(O,OH)_6_, where eight-coordinated *A* is a rare earth element, Ca, Th or Fe, and six-coordinated *B* is Ti, Nb, Ta or W. The general structural feature of nioboaeschynite-(Ce) resembles that of the other members of the aeschynite group. It is characterized by edge-sharing dimers of [(Nb,Ti)O_6_] octa­hedra which share corners to form a three-dimensional framework, with the *A* sites located in channels parallel to the *b* axis. The average *A*—O and *B*—O bond lengths in nioboaeschynite-(Ce) are 2.471 and 1.993 Å, respectively. Moreover, another eight-coordinated site, designated as the *C* site, is also located in the channels and is partially occupied by *A*-type cations. Additionally, the refinement revealed a splitting of the *A* site, with Ca displaced slightly from Ce (0.266 Å apart), presumably resulting from the crystal-chemical differences between the Ce^3+^ and Ca^2+^ cations.

## Related literature

For background on the aeschynite mineral group, see: Zhabin *et al.* (1960 ▶); Aleksandrov (1962 ▶); Jahnberg (1963 ▶); Fauquier & Gasperin (1970 ▶); Ewing & Ehlmann (1975 ▶); Rosenblum & Mosier (1975 ▶); Giuseppetti & Tadini (1990 ▶); Bonazzi & Menchetti (1999 ▶); Yang *et al.* (2001 ▶); Golobic *et al.* (2004 ▶); Ercit (2005 ▶); Škoda & Novák (2007 ▶); Thorogood *et al.* (2010 ▶). For studies on the semiconducting properties of compounds with aeschynite-type structures, see: Kan & Ogawa (2008 ▶); Sumi *et al.* (2010 ▶). For studies of phospho­rescent compounds with aeschynite-type structures, see: Ma *et al.* (2007 ▶); Qi *et al.* (2010 ▶). For information on ionic radii, see: Shannon (1976 ▶).

## Experimental

### 

#### Crystal data

Ca_0.25_Ce_0.79_(Nb_1.14_Ti_0.86_)O_6_
*M* *_r_* = 363.83
Orthorhombic, 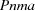

*a* = 11.0563 (15) Å
*b* = 7.560 (1) Å
*c* = 5.3637 (7) Å
*V* = 448.33 (10) Å^3^
*Z* = 4
Mo *K*α radiation
μ = 12.06 mm^−1^
*T* = 293 K
0.06 × 0.06 × 0.05 mm

#### Data collection

Bruker APEXII CCD area-detector diffractometer
Absorption correction: multi-scan (*SADABS*; Sheldrick, 2005 ▶) *T*_min_ = 0.532, *T*_max_ = 0.584
3666 measured reflections
883 independent reflections
737 reflections with *I* > 2σ(*I*)
*R*_int_ = 0.026

#### Refinement

*R*[*F* ^2^ > 2σ(*F* ^2^)] = 0.023
*wR*(*F* ^2^) = 0.055
*S* = 1.09
883 reflections
55 parameters
2 restraints
Δρ_max_ = 2.07 e Å^−3^
Δρ_min_ = −0.80 e Å^−3^

### 

Data collection: *APEX2* (Bruker, 2004 ▶); cell refinement: *SAINT* (Bruker, 2004 ▶); data reduction: *SAINT*; program(s) used to solve structure: *SHELXS97* (Sheldrick, 2008 ▶); program(s) used to refine structure: *SHELXL97* (Sheldrick, 2008 ▶); molecular graphics: *XtalDraw* (Downs & Hall-Wallace, 2003 ▶); software used to prepare material for publication: *publCIF* (Westrip, 2010 ▶).

## Supplementary Material

Crystal structure: contains datablock(s) I, global. DOI: 10.1107/S1600536812031765/wm2645sup1.cif

Structure factors: contains datablock(s) I. DOI: 10.1107/S1600536812031765/wm2645Isup2.hkl

Additional supplementary materials:  crystallographic information; 3D view; checkCIF report

## Figures and Tables

**Fig. 1.**
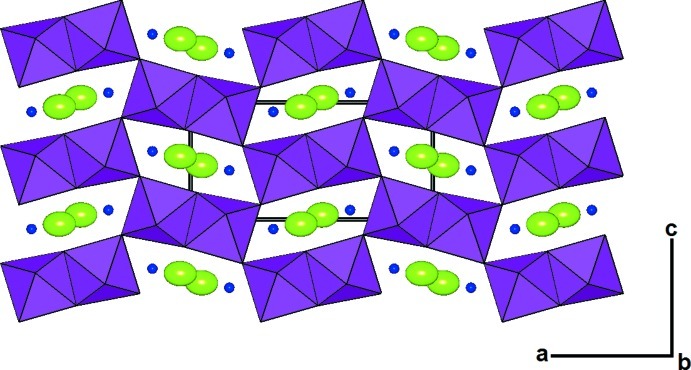
The crystal structure of nioboaeschynite-(Ce). Purple octahedra and small blue spheres (with arbitrary radius) represent the [(Nb,Ti)O6] groups and C-site cations, respectively. Large green displacement ellipsoids at the 99% probability level represent the A-site cations.

**Fig. 2.**
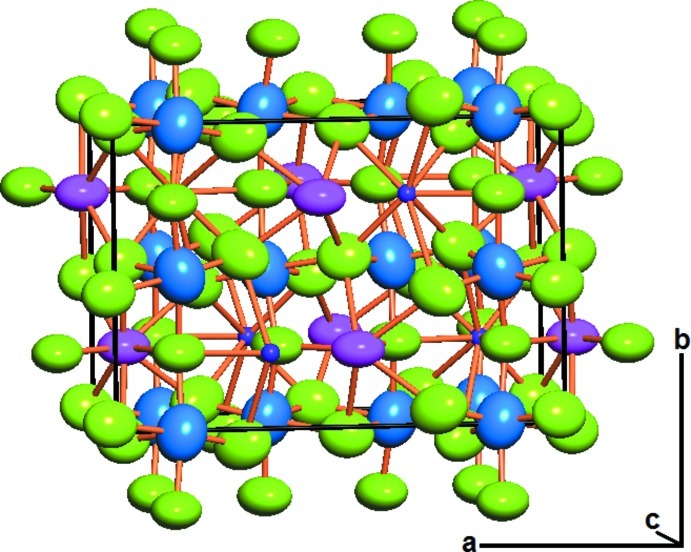
The crystal structure of nioboaeschynite-(Ce) represented with displacement ellipsoids at the 99% probability level. Blue, purple and green ellipsoids represent (Nb,Ti), A-site Ce, and O atoms, respectively. Purple spheres, with arbitrary radius, represent C-site Ce atoms. For clarity, the A-site splitting is not shown.

**Fig. 3.**
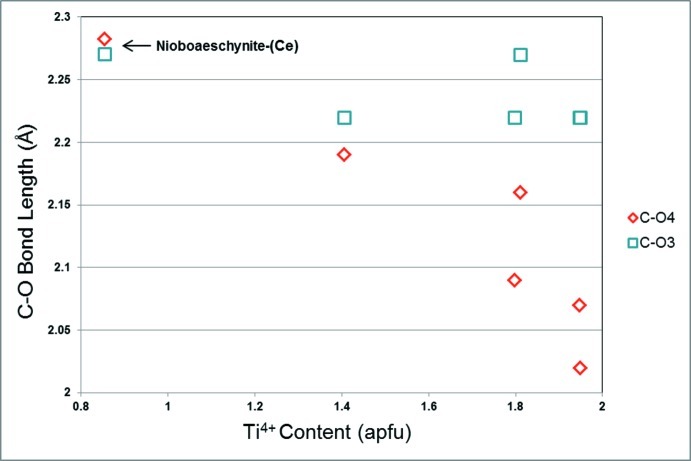
Variations of the two shortest C—O bond lengths with the Ti content in the C-site of aeschynite-(Y) and nioboaeschynite-(Ce). Nioboaeschynite-(Ce) data points are from this study and all other data points for aeschynite-(Y) are taken from Bonazzi & Menchetti (1999).

## supplementary crystallographic information

### Comment

Minerals of the aeschynite group exhibit the CaTa_2_O_6_-structure type with space group *Pnma* and *Z* = 4. They can be characterized by the general formula *AB*_2_(O,OH)_6_, where 8-coordinated *A* is a rare earth element (*REE*), Ca, Th, Fe, and 6-coordinated *B* is Ti, Nb, Ta, W. There are eight members of this group in the current list of minerals approved by the International Mineralogical Association (IMA), including aeschynite-(Ce) (Ce,Ca,Fe,Th)(Ti,Nb)_2_(O,OH)_6_, aeschynite-(Nd) Nd(Ti,Nb)_2_(O,OH)_6_, aeschynite-(Y) (Y,Ca,Fe,Th)(Ti,Nb)_2_(O,OH)_6_, nioboaeschynite-(Ce) (Ce,Ca)(Nb,Ti)_2_(O,OH)_6_, nioboaeschynite-(Y) (Y,*REE*,Ca,Th,Fe)(Nb,Ti,Ta)_2_(O,OH)_6_, tantalaeschynite-(Y) Y(Ta,Ti,Nb)_2_O_6_, vigezzite (Ca,Ce)(Nb,Ta,Ti)_2_O_6_ and rynersonite CaTa_2_O_6_. Aeschynite-type materials have been the subject of numerous investigations for their industrial and scientific importance, for example, as phosphors (Ma *et al.*, 2007; Qi *et al.*, 2010) and as semiconductors for their microwave dielectric properties in ceramics (Kan & Ogawa, 2008; Sumi *et al.*, 2010). There have been a number of structure studies on synthetic aeschynite-group materials, such as CaTa_2_O_6_ (Jahnberg, 1963), LaNbTiO_6_ (Fauquier & Gasperin, 1970; Golobic *et al.*, 2004), and *REE*TiTaO_6_ (*REE* = La, Ce, Pr, Nd, Sm, Eu, Gd, Tb, Dy, Ho, Er, Tm, Yb and Lu) (Thorogood *et al.*, 2010). However, due to prevalent metamictization in natural samples, only the crystal structures of aeschynite-(Ce) (Aleksandrov, 1962), aeschynite-(Y) (Bonazzi & Menchetti, 1999), vigezzite (Giuseppetti & Tadini, 1990), and rynersonite (Jahnberg, 1963) have been reported thus far. Among them, the structure of aeschynite-(Y) is of particular interest, because, besides the *A* and *B* sites, an additional, partially occupied cation site, designated as the *C-* site, was observed (Bonazzi & Menchetti, 1999). The coordination environment of the *C-*site in this mineral is similar to that of the *A-* site, but the shortest *C*—O bond length (*C*—O4) is only ~2.10 Å, similar to that of the *B*—O bonds. As all five natural aeschynite-(Y) samples examined by Bonazzi & Menchetti (1999) contain excess *B*-type cations (*B* > 2.0 atoms per formula unit; apfu) and are deficient in *A*-type cations (*A* < 1.0 apfu) with respect to the ideal chemical formula, a *B*-type cation (W) was thus assigned to the *C*-site. Yet, due to the close proximity of the *A*- and *C*-sites (~2.5 Å apart), Bonazzi & Menchetti (1999) assumed that the occupancy of the *C*-site is coupled with a vacancy in the *A*-site, giving rise to the structure formula *A*_1-_*_x_**B*_2_*C_x_*(O,OH)_6_.

Nioboaeschynite-(Ce) from the Vishnevy Mountains, Russia was first described by Zhabin *et al.* (1960) and later from the Tanana quadrangle, central Alaska by Rosenblum & Mosier (1975). In both studies unit-cell parameters were determined, but not the crystal structures. Owing to its metamict nature, subsequent studies involving nioboaeschynite-(Ce) were mainly focused on chemical variations within the group and compositional trends between the aeschynite group and the closely-related euxenite group (Ewing & Ehlmann, 1975; Yang *et al.*, 2001; Ercit, 2005; Škoda & Novák, 2007). Notably, the aeschynite-(Ce) sample used in the structure refinement by Aleksandrov (1962) contained 50.5% Nb and 49.5% Ti, thus making it effectively nioboaeschynite-(Ce), according to current IMA nomenclature. Regardless, the structure of this mineral was only determined on the basis of photographic intensity data with *R* = 12.5%. In the course of identifying minerals for the RRUFF project (http://rruff.info), we found a well crystallized nioboaeschynite-(Ce) sample from the Upper Fir carbonatite, Kamloops mining division, British Columbia, Canada and determined its structure by means of single-crystal X-ray diffraction.

The structure of nioboaeschynite-(Ce) is very similar to that of the aeschynite-(Y) reported by Bonazzi & Menchetti (1999), including the presence of an additional, partially occupied *C*-site. The general structural feature of nioboaeschynite-(Ce) are edge-sharing dimers of [(Nb,Ti)O_6_] octahedra that share corners to form a three-dimensional framework, with the 8-coordinated *A*- and *C*-sites located in the channels running parallel to the *b* axis (Figs. 1,2). The average *A*—O, *B*—O, and *C*—O bond lengths are 2.471, 1.993, and 2.474 Å, respectively, which are all longer than the corresponding ones (~2.393, 1.979, and 2.39 Å) in aeschynite-(Y) (Bonazzi & Menchetti, 1999). Interestingly, the shortest bond length within the [*C*O_8_] polyhedron is the *C*—O4 bond in aeschynite-(Y) (~2.11 Å) (Bonazzi & Menchetti, 1999), whereas it is *C*—O3 in nioboaeschynite-(Ce) [2.27 (1) Å]. This difference appears to correlate with the increase in the *C*—O4 distance associated with decreasing Ti content (or increasing Nb and Ta content) in the *B*-site, while the *C*—O3 bond length is essentially invariable with Ti content (Fig. 3). In this study, we assigned some *A*-type cations to the *C*-site, because (1) the shortest *C*—O bond in our specimen is significantly longer than that in aeschynite-(Y) (Bonazzi & Menchetti, 1999) and (2) our sample contains excess *A*-type cations, rather than excess *B*-type cations, as in the aeschynite-(Y) samples analyzed by Bonazzi & Menchetti (1999). Accordingly, we propose the structural formula *AB*_2_*C_x_*O_6_ for the nioboaeschynite-(Ce) from the Upper Fir carbonatite. Our results, coupled with that of the Bonazzi & Menchetti (1999) study, indicate that there is great flexibility in the formula of the aeschynite groups minerals due to the occupancy variations permitted by the *C*-site. Furthermore, we detected a splitting of the *A*-site in our refinement, with Ca displaced slightly from Ce (0.266 Å apart). Although this site splitting may be related to the presence of some *A*-type cations in the *C*-site to minimize the cation-cation repulsion due to the short *A—C* distance (~2.4 Å), a 25% occupancy of *A*' by Ca does not agree with the 3.8% occupancy of *C*. The observed site splitting in our sample is, therefore, more likely a result of the different crystal-chemical behavior of the Ce^3+^ and Ca^2+^ cations.

From a mineralogical point of view, ideal chemical formulas are treated differently from those reported for synthetic compounds by chemists. There are no two grains of a mineral that will have exactly the same measured chemical composition; therefore, the ideal chemical formula of a mineral, as defined by the IMA, comes with understood tolerances. Ideal formulas are necessary to distinguish and designate one mineral species from another. In the case of nioboaeschynite-(Ce), the current IMA formula is (Ce,Ca)(Nb,Ti)_2_(O,OH)_6_, where we understand Ca, Ti, OH to be minor chemical components. An ideal formula given in this format has two possible meanings. One is that the Ca substitution at the Ce-containing *A*-site is minor, but essential to constrain the mineral into its observed crystal structure, as likewise for Ti at the Nb site, and OH is variable to account for charge balance. The other possibility is that the original workers described the formula this way because, while they could not decide if the minor elements were essential or not, the minor elements were common enough that they listed them in the formula anyway. However, the structural studies on synthetic aeschynite group crystals, including *REE*TiTaO_6_ (*REE* = La, Ce, Pr, Nd, Sm, Eu, Gd, Tb, Dy, Ho, Er, Tm, Yb and Lu) compounds (Thorogood *et al.*, 2010) and LaNbTiO_6_ (Fauquier & Gasperin, 1970; Golobic *et al.* 2004), prove that the aeschynite structure is stable in the complete absence of Ca, and with an ideal 1:1 ratio of Ti:(Ta,Nb). Furthermore, because Nb^5+^ and Ta^5+^ have the same charge, the same ionic radius of 0.64 Å (Shannon, 1976), and exhibit similar chemical behavior, they can substitute for each other without affecting the Ti content (Škoda & Novák, 2007). Therefore, it seems reasonable to consider that the ideal nioboaeschynite-(Ce) chemical formula should be the charge-balanced Ce(NbTi)O_6_, with (Nb,Ti) variations charge-balanced by variations in *A*-site chemistry, such as Ca^2+^ or Th^4+^. The modified ideal formula, Ce(NbTi)O_6_, however, is problematic because it is likely the same ideal formula is applicable to aeschynite-(Ce), Ce(TiNb)O_6_ (Aleksandrov, 1962). The two minerals are unnecessarily distinguished by the dominant cation at the *B*-site.

### Experimental

The nioboaeschynite-(Ce) specimen used in this study is from the Upper Fir carbonatite, Kamloops mining division, British Columbia, Canada and is in the collection of the RRUFF project (deposition No. R110056; http://rruff.info). The chemical composition was measured with a CAMECA SX100 electron microprobe at the conditions of 25 keV, 20 nA, and a beam size of 10 µm. An average of 26 analysis points yielded (wt. %): P_2_O_5_ 0.02, CaO 3.72, TiO_2_ 18.38, FeO 0.59, SrO 0.18, Nb_2_O_5_ 40.12, Y_2_O_3_ 0.52, La_2_O_3_ 5.39, Ce_2_O_3_ 15.25, Pr_2_O_3_ 1.79, Nd_2_O_3_ 6.29, SmO 1.02, Gd_2_O_3_ 1/2, Ta_2_O_5_ 0.07, WO_3_ 0.06, PbO 0.03, ThO_2_ 4.08, UO_2_ 0.22. The empirical chemical formula, calculated based on 6 O atoms, is (Ce_0.35_Nd_0.14_La_0.12_Pr_0.04_Sm_0.02_Y_0.02_Gd_0.01_Ca_0.25_Th_0.06_ Fe_0.03_Sr_0.01_)_Σ__=1.05_ (Nb_1.14_Ti_0.85_)_Σ__=1.99_O_6_. The formula is charge balanced and there is no evidence of OH in the sample's Raman spectra or structural analysis.

### Refinement

During the structure refinement, due to similar X-ray scattering lengths, all rare earth elements were treated as Ce. A preliminary refinement revealed the presence of some cations in the *C*-site. Since our sample contains excess *A*-type cations (0.05 apfu), we subsequently refined the occupancy of the *C*-site using the scattering factors of Ce with an isotropic displacement parameter, which reduced the *R*1 factor from 0.0313 to 0.0281 and yielded a site occupancy of 0.04 Ce apfu. However, because of the chemical complexity of our sample, it is difficult to determine exactly what element(s) preferentially reside(s) in the *C*-site. According to the refinement, the *C*-site contains approximately 2.22 electrons. Based on the electron microprobe chemistry data, (Ce_0.35_Nd_0.14_La_0.12_Pr_0.04_Sm_0.02_Y_0.02_Gd_0.01_Ca_0.25_Th_0.06_ Fe_0.03_Sr_0.01_)_Σ__=1.05_(Nb_1.14_Ti_0.85_)_Σ__=1.99_O_6_, there is no single element whose abundance would supply the *C*-site with the required number of electrons. However, the electrons supplied by a combination of *REE* and Th from the excess 0.05 atoms in the *A*-site, based on their respective abundances, is approximately 2.30. Worth noting is that if the excess 0.05 atoms were designated to be Ca, only one electron would be allotted to the *C*-site. Additionally, while the average *C*-site bond length does correspond to that of Ca—O, it also corresponds to that of the average bond length of (*REE* + Th). The average (8-coordinated) Ca^2+^ ionic radius is 1.12 Å (Shannon, 1976) and the average (*REE* + Th) ionic radius is 1.126 Å (based on their abundances as determined by the microprobe chemistry data and their radii by Shannon, 1976). Therefore, Ce was chosen to represent (*REE* + Th) in the *C*-site. Moreover, from difference Fourier synthesis, we noticed a significant, positive residual peak that is ~0.2 Å from the *A*-site. An *A*-site splitting model was then assumed, with Ce occupying the *A*-site and Ca occupying the *A*'-site, which led to a further reduction of the *R*1 factor from 0.0281 to 0.0234. The refined occupancies are ~0.75 for the *A*-site and ~0.25 for the *A*'-site, matching the measured chemical component of Ca remarkably. In the final refinement, we assumed that the *A*- and *B*-sites are fully occupied by Ce/Ca and Nb/Ti, respectively, and their ratios were constrained to those determined from the electron microprobe analysis. Because of the strong correlation in the displacement parameters between the *A*- and *A*'-sites and the low occupancy at the *C*-site, only isotropic displacement parameters were refined for the *A*'- and *C*-sites. The highest residual peak in the difference Fourier maps was located at (0.5269, 1/4, 0.0261), 0.77 Å from the *A*-site, and the deepest hole at (0.3258, 0.7007, 0.0253), 0.50 Å from the *C*-site.

### Crystal data

**Table:**

Ca_0.25_Ce_0.79_(Nb_1.14_Ti_0.86_)O_6_	*F*(000) = 650
*M**_r_* = 363.83	*D*_x_ = 5.315 Mg m^−^^3^
Orthorhombic, *P**n**m**a*	Mo *K*α radiation, λ = 0.71073 Å
Hall symbol: -P 2ac 2n	Cell parameters from 1243 reflections
*a* = 11.0563 (15) Å	θ = 4.6–32.6°
*b* = 7.560 (1) Å	µ = 12.06 mm^−^^1^
*c* = 5.3637 (7) Å	*T* = 293 K
*V* = 448.33 (10) Å^3^	Tabular, metallic gray
*Z* = 4	0.06 × 0.06 × 0.05 mm

### Data collection

**Table:**

Bruker APEXII CCD area-detector diffractometer	883 independent reflections
Radiation source: fine-focus sealed tube	737 reflections with *I* > 2σ(*I*)
Graphite monochromator	*R*_int_ = 0.026
φ and ω scan	θ_max_ = 32.8°, θ_min_ = 4.2°
Absorption correction: multi-scan (*SADABS*; Sheldrick, 2005)	*h* = −15→16
*T*_min_ = 0.532, *T*_max_ = 0.584	*k* = −11→4
3666 measured reflections	*l* = −8→8

### Refinement

**Table:**

Refinement on *F*^2^	2 restraints
Least-squares matrix: full	Primary atom site location: structure-invariant direct methods
*R*[*F*^2^ > 2σ(*F*^2^)] = 0.023	Secondary atom site location: difference Fourier map
*wR*(*F*^2^) = 0.055	*w* = 1/[σ^2^(*F*_o_^2^) + (0.0239*P*)^2^ + 1.525*P*] where *P* = (*F*_o_^2^ + 2*F*_c_^2^)/3
*S* = 1.09	(Δ/σ)_max_ = 0.001
883 reflections	Δρ_max_ = 2.07 e Å^−^^3^
55 parameters	Δρ_min_ = −0.80 e Å^−^^3^

### Special details

**Table:**

Geometry. All e.s.d.'s (except the e.s.d. in the dihedral angle between two l.s. planes) are estimated using the full covariance matrix. The cell e.s.d.'s are taken into account individually in the estimation of e.s.d.'s in distances, angles and torsion angles; correlations between e.s.d.'s in cell parameters are only used when they are defined by crystal symmetry. An approximate (isotropic) treatment of cell e.s.d.'s is used for estimating e.s.d.'s involving l.s. planes.
Refinement. Refinement of *F*^2^ against ALL reflections. The weighted *R*-factor *wR* and goodness of fit *S* are based on *F*^2^, conventional *R*-factors *R* are based on *F*, with *F* set to zero for negative *F*^2^. The threshold expression of *F*^2^ > σ(*F*^2^) is used only for calculating *R*-factors(gt) *etc*. and is not relevant to the choice of reflections for refinement. *R*-factors based on *F*^2^ are statistically about twice as large as those based on *F*, and *R*- factors based on ALL data will be even larger.

### Fractional atomic coordinates and isotropic or equivalent isotropic displacement parameters (Å^2^)

**Table:**

	*x*	*y*	*z*	*U*_iso_*/*U*_eq_	Occ. (<1)
CeA	0.45727 (9)	0.2500	0.03835 (14)	0.00893 (11)	0.7500 (1)
CaA'	0.4338 (9)	0.2500	0.050 (2)	0.018 (3)*	0.2500 (1)
NbB	0.35726 (3)	0.50690 (5)	0.53830 (8)	0.01227 (11)	0.5700 (1)
TiB	0.35726 (3)	0.50690 (5)	0.53830 (8)	0.01227 (11)	0.4300 (1)
O1	0.2875 (2)	0.4417 (3)	0.8720 (5)	0.0126 (5)	
O2	0.5259 (2)	0.4615 (3)	0.7310 (4)	0.0105 (4)	
O3	0.6221 (3)	0.2500	0.3389 (7)	0.0124 (7)	
O4	0.3560 (3)	0.2500	0.4526 (7)	0.0123 (6)	
CeC	0.1586 (16)	0.2500	0.578 (3)	0.063 (6)*	0.038 (2)

### Atomic displacement parameters (Å^2^)

**Table:**

	*U*^11^	*U*^22^	*U*^33^	*U*^12^	*U*^13^	*U*^23^
CeA	0.0122 (3)	0.0059 (2)	0.0087 (2)	0.000	0.0005 (2)	0.000
NbB	0.01110 (19)	0.01448 (19)	0.01123 (19)	0.00121 (13)	−0.00065 (13)	0.00082 (14)
TiB	0.01110 (19)	0.01448 (19)	0.01123 (19)	0.00121 (13)	−0.00065 (13)	0.00082 (14)
O1	0.0133 (11)	0.0109 (10)	0.0137 (12)	0.0014 (9)	0.0041 (9)	0.0014 (10)
O2	0.0127 (10)	0.0096 (10)	0.0091 (11)	−0.0004 (8)	0.0016 (8)	0.0005 (9)
O3	0.0114 (15)	0.0062 (14)	0.0195 (19)	0.000	0.0016 (13)	0.000
O4	0.0123 (15)	0.0066 (14)	0.0179 (18)	0.000	0.0016 (13)	0.000

### Geometric parameters (Å, º)

**Table:**

CeA—O2^i^	2.418 (2)	CaA'—O3	2.596 (12)
CeA—O2^ii^	2.418 (2)	NbB—O1^v^	1.873 (2)
CeA—O3	2.433 (4)	NbB—O2^iii^	1.953 (2)
CeA—O4	2.488 (4)	NbB—O3^iii^	1.9655 (14)
CeA—O2^iii^	2.515 (2)	NbB—O4	1.9959 (10)
CeA—O2^iv^	2.515 (2)	NbB—O1	2.011 (3)
CeA—O1^i^	2.534 (3)	NbB—O2	2.159 (2)
CeA—O1^ii^	2.534 (3)	CeC—O3^vi^	2.270 (19)
CaA'—O4	2.327 (12)	CeC—04	2.282 (18)
CaA'—O1^i^	2.372 (9)	CeC—O2^vii^	2.401 (13)
CaA'—O1^ii^	2.372 (9)	CeC—O2^viii^	2.401 (13)
CaA'—O2^iii^	2.519 (6)	CeC—01	2.573 (16)
CaA'—O2^iv^	2.519 (6)	CeC—O1^ix^	2.573 (15)
CaA'—O2^i^	2.551 (9)	CeC—O1^x^	2.647 (9)
CaA'—O2^ii^	2.551 (9)	CeC—O1^v^	2.647 (9)
			
O1^v^—NbB—O2^iii^	100.81 (11)	O3^iii^—NbB—O1	88.61 (13)
O1^v^—NbB—O3^iii^	93.71 (13)	O4—NbB—O1	87.91 (13)
O2^iii^—NbB—O3^iii^	93.23 (13)	O1^v^—NbB—O2	177.17 (10)
O1^v^—NbB—O4	94.95 (13)	O2^iii^—NbB—O2	78.59 (11)
O2^iii^—NbB—O4	87.34 (13)	O3^iii^—NbB—O2	83.57 (12)
O3^iii^—NbB—O4	171.06 (15)	O4—NbB—O2	87.80 (12)
O1^v^—NbB—O1	98.46 (6)	O1—NbB—O2	82.32 (10)
O2^iii^—NbB—O1	160.48 (10)		

Symmetry codes: (i) *x*, −*y*+1/2, *z*−1; (ii) *x*, *y*, *z*−1; (iii) −*x*+1, −*y*+1, −*z*+1; (iv) −*x*+1, *y*−1/2, −*z*+1; (v) −*x*+1/2, −*y*+1, *z*−1/2; (vi) *x*−1/2, *y*, −*z*+1/2; (vii) *x*−1/2, −*y*+1/2, −*z*+3/2; (viii) *x*−1/2, *y*, −*z*+3/2; (ix) *x*, −*y*+1/2, *z*; (x) −*x*+1/2, *y*−1/2, *z*−1/2.
